# Correlation of physical and cognitive impairment in diabetic and hypertensive frail older adults

**DOI:** 10.1186/s12933-021-01442-z

**Published:** 2022-01-19

**Authors:** Pasquale Mone, Jessica Gambardella, Angela Lombardi, Antonella Pansini, Stefano De Gennaro, Anna Luisa Leo, Michele Famiglietti, Anna Marro, Maria Morgante, Salvatore Frullone, Antonio De Luca, Gaetano Santulli

**Affiliations:** 1 Department of Medicine, Einstein Institute for Aging Research, Einstein-Mount Sinai Diabetes Research Center (ES-DRC), The Fleischer Institute for Diabetes and Metabolism (FIDAM), Albert Einstein College of Medicine, New York, NY USA; 2 Department of Mental and Physical Health and Preventive Medicine, University of Campania “Luigi Vanvitelli”, Naples, Italy; 3 Division of Internal Medicine, Sant’Angelo Dei Lombardi Hospital, ASL (Local Health Unit), Avellino, Italy; 4 International Translational Research and Medical Education (ITME) Consortium, Department of Advanced Biomedical Sciences, “Federico II” University, Naples, Italy; 5 Elderly Assistance and Home Care, ASL (Local Health Unit), Avellino, Italy; 6 Department of Molecular Pharmacology, Institute for Neuroimmunology and Inflammation (INI), Wilf Family Cardiovascular Research Institute, Albert Einstein College of Medicine, New York, NY USA

**Keywords:** Diabetes, Hypertension, Frailty, Cognitive impairment, Physical impairment

## Abstract

**Background:**

Diabetes and hypertension are common in older adults and represent established risk factors for frailty. Frailty is a multidimensional condition due to reserve loss and susceptibility to stressors with a high risk of death, hospitalizations, functional and cognitive impairment. Comorbidities such as diabetes and hypertension play a key role in increasing the risk of mortality, hospitalization, and disability. Moreover, frail patients with diabetes and hypertension are known to have an increased risk of cognitive and physical impairment. Nevertheless, no study assessed the correlation between physical and cognitive impairment in frail older adults with diabetes and hypertension.

**Methods:**

We evaluated consecutive frail older patients with diabetes and hypertension who presented at ASL (local health unit of the Italian Ministry of Health) Avellino, Italy, from March 2021 to October 2021. The inclusion criteria were: a previous diagnosis of diabetes and hypertension with no evidence of secondary causes; age > 65 years; a frailty status; Montreal Cognitive Assessment (MoCA) score < 26.

**Results:**

179 patients successfully completed the study. We found a strong and significant correlation between MoCA score and 5-m gait speed test (r: 0.877; p < 0.001). To further verify our results, we performed a linear multivariate analysis adjusting for potential confounding factors, with MoCA score as dependent variable, which confirmed the significant association with glycemia (p < 0.001).

**Conclusions:**

This is the first study showing a significant correlation between 5-m gait speed test and MoCA score in frail diabetic and hypertensive older adults.

## Background

Hypertension and Type 2 Diabetes Mellitus (herein called diabetes) are very common in older adults [1–8]. Furthermore, both disorders are well-known risk factors for frailty [9–14], a multidimensional condition due to reserve loss and susceptibility to stressors with a high risk of death, hospitalizations, functional and cognitive impairment [15–19]. Evaluating and properly treating comorbidities and complications is crucial to reduce the incidence of cognitive and physical impairment; hence, clinical evaluation is the main goal to obtain an early diagnosis and a timely treatment to prevent adverse events [20–26]. Of note, frail patients with diabetes and hypertension are known to have a higher risk of cognitive and physical impairment [27–34]. Nevertheless, no report hitherto evaluated the actual correlations between physical and cognitive impairment in frail older adults with diabetes and hypertension. Our study, thus, aimed to investigate the relationships between physical and cognitive impairment in this previously reported population.

## Methods

We recruited consecutive frail older patients with diabetes and hypertension from March 2021 to October 2021 at ASL (local health unit of the Italian Ministry of Health) Avellino, Italy. Inclusion criteria were: age > 65 years; a previous diagnosis of diabetes and hypertension with no evidence of secondary causes; a frailty status; Montreal Cognitive Assessment (MoCA) score < 26. Exclusion Criteria were: Age < 65 years; absence of frailty status; absence of diabetes and hypertension; previous cerebrovascular events; left ventricular ejection fraction < 25%, with previous myocardial infarction or previous PPCI and/or coronary artery by-pass grafting.

All patients underwent blood pressure measurement, heart rate (HR) evaluation, and blood analysis to assess glycemia and HbA1c. An informed consent was signed by each patient (or legal representative). Research was performed according to the 1975 Declaration of Helsinki and its later amendments. The Institutional Review Board of Campania Nord approved the protocol.

### Assessment of cognitive function

Global cognitive function was assessed via MoCA test. This cognitive test covers many cognitive skills, scores range from 0 to 30, and cognitive impairment is defined by values < 26. This test assesses the main cognitive areas: immediate and delayed memory (free and cued recall), language, visuoperceptual and visuospatial capacities, motor planning, executive function, attention, and cognitive judgment. Instead, MMSE scores are influenced by demographic variables such as age and years of education: subjects with higher education levels have better results than subjects with lower levels. In particular, older adults show worst performances in MMSE scores that are age-dependent [35–37]. MoCA test is more specific to evaluate cognitive domains (attention, concentration, memory, language, calculation, orientation and executive functions) and is considered the best test to detect mild cognitive impairment [38, 39].

### Frailty evaluation

A physical frailty assessment was performed following the Fried Criteria, as previously described [23, 24]. A diagnosis of frailty status was made with at least three points out of five, whereas patients having one or two points were considered pre-frails and, as such, excluded:

-Low physical activity level (a weighted score of kilocalories expended per week was calculated at baseline based on each participant’s report. The lowest quintile of physical activity was identified for each gender).
-Weight loss (unintentional loss ≥ 4.5 kg in the past year).
-Exhaustion (poor endurance and energy, self-reported). Self-reported exhaustion, identified by two questions from the CES–D scale, is associated with stage of exercise reached in graded exercise testing, as an indicator of O_2_ max, and is predictive of cardiovascular disease.
-Weakness (handgrip strength in the lowest 20% quintile at baseline, adjusted for sex and body mass index).
-Slowness (walking speed under the lowest quintile adjusted for sex and height).

Additionally, we performed a 5-m gait speed test in all patients before discharge. This test is among the most used approaches to measure the time required to walk a short distance at a comfortable pace; an altered gait speed test has been associated with impairments in lower-extremity muscle function, as well as neurosensory and cardiopulmonary dysfunction [40, 41]. Previous reports have shown that performing a 5-m gait speed test alone is sufficient to evaluate the frailty status in patients with cardiovascular diseases [40, 42–45].

### Statistical analysis

Data are presented as mean ± SD. Based on our preliminary findings in a pilot study (rho: 0.26), we calculated the number of patients required for the study to reject the null hypothesis 95% of the time (*i.e*., with a one-tailed type II error rate of 0.05) with a two-tailed type I error at the 0.05 level of significance; the sample size was calculated via GPOWER software, yielding a minimum size of 151 patients. We applied a dispersion model correlating MoCA score and 5-m gait speed test; we also performed a linear regression analysis with MoCA score as dependent variable adjusting for potential confounding factors, including age, sex, BMI, blood pressure, HR, glycemia, HbA1c, and comorbidities. All calculations were computed using the SPSS 26 software.

## Results

**Fig. 1:**
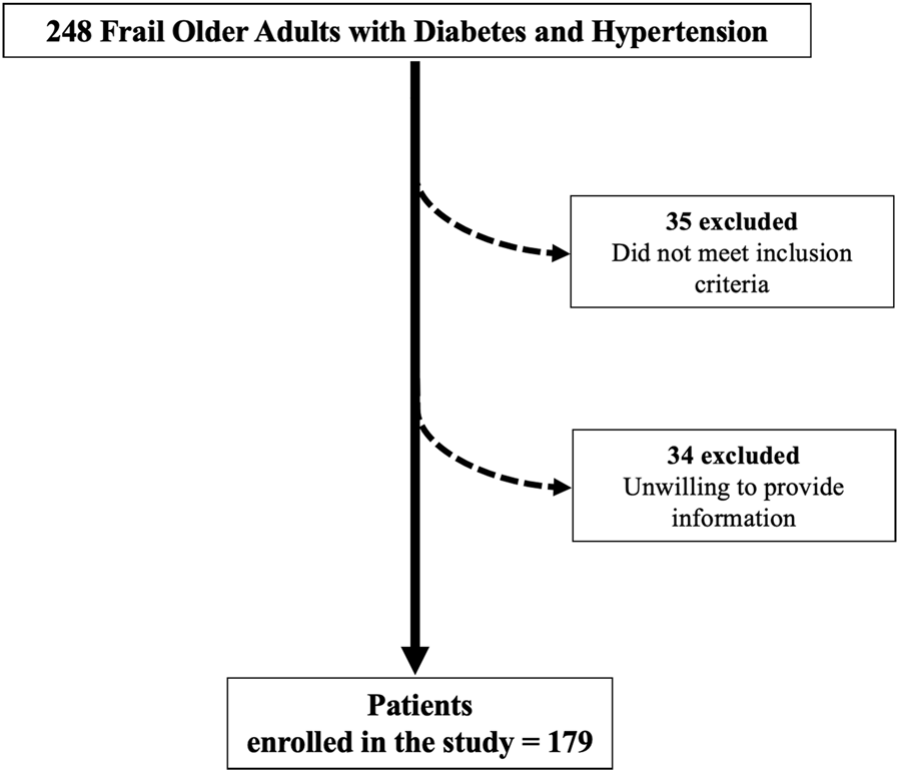
Study flow chart

We evaluated 248 frail elders with diabetes and hypertension. Since 34 patients were unwilling to provide clinical information, and 35 subjects did not meet inclusion criteria, 179 patients met the inclusion and exclusion criteria (Fig. 1). The clinical characteristics of our study group are reported in Table 1. There were no significant differences in age, BMI, sex distribution, smoking habits, are reported in between the two groups (Table 1).

**Table 1:** Clinical characteristics of the patients

	Values
N	179
Sex (M/F)	74/105
Mean age (years)	81.0 ± 8.5
BMI (kg/m^2^)	28.5 ± 1.4
SBP (mmHg)	129.3 ± 11.7
DBP (mmHg)	77.5 ± 9.5
Heart rate (bpm)	81.0 ± 9.0
5mGS test (m/s)	0.6 ± 0.1
Comorbidities	
COPD	55 (30.7)
CKD	64 (35.8)
HF	66 (37.5)
Hyperlipidemia	70 (39.1)
Laboratory analyses	
Plasma glucose (mg/dl)	166.0 ± 58.67
HbA1c (mmol/l)	7.5 ± 0.7
Global cognitive function	
MoCA	20.59 ± 3.8
Fried Criteria	
Weight loss	130 (72.6)
Exhaustion	57 (31.9)
Low physical activity	55 (30.7)
Slowness	141 (78.8)
Weakness	154 (86.0)

Data are means ± SD for continuous variables or n (%) for categorical variables

*5mGS* 5-meter gait speed, *BMI* body mass index, *CKD* chronic kidney disease, *COPD* chronic obstructive pulmonary disease, *DBP* diastolic blood pressure, *HbA1c* glycated hemoglobin, *HF* heart failure, *MoCA* Montreal Cognitive Assessment, *SBP* systolic blood pressure

Concerning comorbidities, which are of particular importance in a population like the one investigated in our study, we detected COPD in 30.7% of patients, CKD in 35.8%, HF in 37.5%,). The use of diuretics, angiotensin-converting enzyme inhibitors, beta-blockers, and calcium blockers was also similar between the two groups (Table 1).

**Fig. 2:**
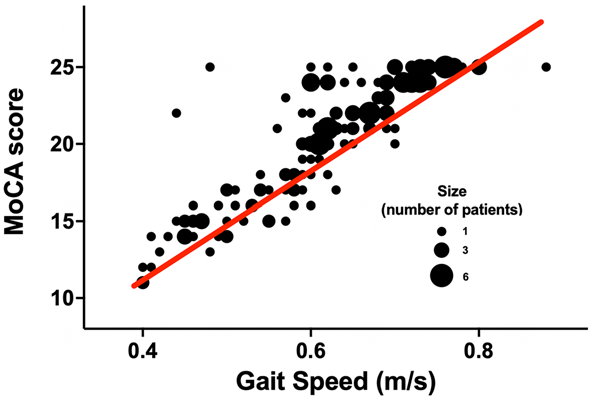
Dispersion model (bubble chart) between MoCA score and gait speed test (r: 0.877; p < 0.001)

We found a significant correlation between MoCA score and 5-m gait speed test (r: 0.877; p < 0.001), as shown in Fig. 2. In the effort to confirm our results, we performed a linear multivariate analysis with MoCA score as the dependent variable, adjusting for potential confounding factors, including age, sex, BMI, blood pressure, HR, glycemia, HbA1c, and comorbidities. We observed (Table 2) a significant association with glycemia and age (p < 0.001); furthermore, we observed significant results for sex (0.002), HR (p: 0.003), HF (p 0.010), and CKD (p 0.022).

**Table 2:** Multivariate Regression Analysis using the MoCA score as the dependent variable

	B	Standard error	Beta	t	p	95% Confidence Interval	
						Lower bound	Upper bound
Age	− 0.126	0.038	− 0.176	− 3.306	< 0.001	− 0.202	− 0.051
Sex	− 1.275	0.402	− 0.165	− 3.171	0.002	− 2.069	− 0.481
BMI	− 0.153	0.117	− 0.068	− 1.312	0.191	− 0.383	0.077
SBP	− 0.049	0.025	− 0.101	− 1.944	0.054	− 0.099	0.001
DBP	0.037	0.030	0.063	1.237	0.218	− 0.022	0.096
HR	0.070	0.023	0.163	3.053	0.003	0.025	0.116
Glycemia	− 0.037	0.004	− 0.568	− 10.291	< 0.001	− 0.044	− 0.030
HbA1c	0.011	0.275	0.002	0.042	0.867	− 0.532	0.555
HF	− 1.081	0.414	− 0.141	− 2.611	0.010	− 1.899	− 0.264
Hyperlipidemia	0.494	0.406	0.063	1.217	0.225	− 0.307	1.295
COPD	0.216	0.479	0.026	0.450	0.653	− 0.730	1.161
CKD	− 1.111	0.480	− 0.139	− 2.316	0.022	− 2.059	− 0.164

*BMI* body mass index, *CKD* chronic kidney disease, *COPD* chronic obstructive pulmonary disease, *DBP* diastolic blood pressure, *Hb1Ac* glycated hemoglobin, *HF* heart failure, *HR* heart rate, *MoCA* Montreal Cognitive Assessment, *SBP* systolic blood pressure

## Discussion

To the best of our knowledge, this study is the first to highlight a strong correlation between physical and cognitive impairment in hypertensive and diabetic elderly patients. Previous studies had highlighted the interaction between physical and cognitive function [46, 47]; however, no study had hitherto investigated this relationship in frail elders with diabetes and hypertension.

The management of frailty in older adults is very debated; comorbidities such as diabetes and hypertension are well recognized to play key roles in increasing the risk of mortality, hospitalization and disability. Indeed, both of them are functionally linked to endothelial dysfunction, inflammation, atherosclerosis, and oxidative stress [48–55] driving cognitive and physical impairment in a complex syndrome such as frailty [56–63].

Our data indicate a primary role of diabetes and hypertension in the development of disability in a frail cohort of older adults. Furthermore, consistent with previous investigations [64, 65], in our population we observed a robust impact of age (p < 0.001) and admission glycemia (p < 0.001), strongly suggesting that glycemic control is a goal to achieve for avoiding adverse outcomes in this class of patients. Indeed, hyperglycemia worsens a delicate balance in patients with multimorbidity such as frail elders [66–68]. Since also female sex had a significant impact in our multivariate analysis, we speculate that frail elderly women with diabetes and hypertension might have a higher risk of atherosclerosis and cardiovascular diseases [69, 70], although this possibility needs to be verified in a dedicated trial.

Taken together, our data suggest that adding a simple evaluation with MoCA and gait speed test may be useful to evaluate cognitive and physical status. We propose to add an assessment of cognitive and physical condition in the comprehensive geriatric evaluation of frail hypertensive diabetic elders. Several limitations deserve consideration. We do not have follow-up records; nonetheless, we believe that observing significant differences is noteworthy, especially in a population of frail older adults. We used a classification of frailty that mainly assesses physical frailty, in contrast to a multidimensional approach also involving nutritional, and psychosocial components. Finally, the sample size of our group is relatively small; however, we had performed an a priori power analysis, based on our preliminary data, showing that the minimum estimated sample size to obtain statistically significant results was 151 patients.

## Conclusions

This study is the first one to correlate MoCA score and 5-m gait speed test in frail diabetic and hypertensive older adults. Further analyses with larger cohorts and a follow-up evaluation are warranted to corroborate our results.

## Data Availability

Data and study materials are available from the First Author upon reasonable request.

## Abbreviations

5mGS: 5-meter gait speed
BMI: Body mass index
CKD: Chronic kidney disease
COPD: Chronic obstructive pulmonary disease
Diabetes: Type 2 diabetes mellitus
DBP: Diastolic blood pressure
Hb1Ac: Glycated hemoglobin
HF: Heart failure
HR: Heart rate
MoCA: Montreal Cognitive Assessment
SBP: Systolic blood pressure
